# Reverse Takotsubo Cardiomyopathy Caused by Repeated Guanfacine Overdose

**DOI:** 10.1016/j.jaccas.2025.105915

**Published:** 2025-11-13

**Authors:** Takayuki Kumaki, Takeshi Kashimura, Sho Hirayama, Hiroki Tsuchiya, Kazuki Deuchi, Takahiro Kanbayashi, Jun Egawa, Hajime Umezu, Kei Nishiyama, Takayuki Inomata

**Affiliations:** a Department of Cardiovascular Medicine, Niigata University Graduate School of Medical and Dental Sciences, Niigata, Japan; b Advanced Disaster Medical and Emergency Critical Care Center, Niigata University Medical and Dental Hospital, Niigata, Japan; c Department of Psychiatry, Niigata University Graduate School of Medical and Dental Sciences, Niigata, Japan; d Division of Pathology, Niigata University Medical and Dental Hospital, Niigata, Japan

**Keywords:** ADHD, guanfacine, reverse takotsubo cardiomyopathy

## Abstract

**Background:**

Guanfacine, an α2A receptor agonist, is prescribed to reduce cerebral neural activity in patients with attention-deficit hyperactivity disorder.

**Case Summary:**

We present the case of a 15-year-old girl who overdosed on extended-release guanfacine 3 times. This resulted in 2 episodes of reverse takotsubo cardiomyopathy with respiratory failure requiring mechanical ventilation, followed by late QT prolongation and spontaneous recovery.

**Discussion:**

The prominent characteristic of this case was the blood guanfacine concentration–dependent development of reverse takotsubo cardiomyopathy, providing insights into the underlying mechanisms.

**Take-Home Messages:**

An overdose of guanfacine, a medication used for treating attention-deficit hyperactivity disorder, can result in reverse takotsubo cardiomyopathy in a blood concentration–dependent manner. The cardiopulmonary time course of an extended-release guanfacine overdose spans approximately 2 weeks, with early critical respiratory failure, reverse takotsubo cardiomyopathy, and late QT prolongation.

## Visual Summary

**Figure:**
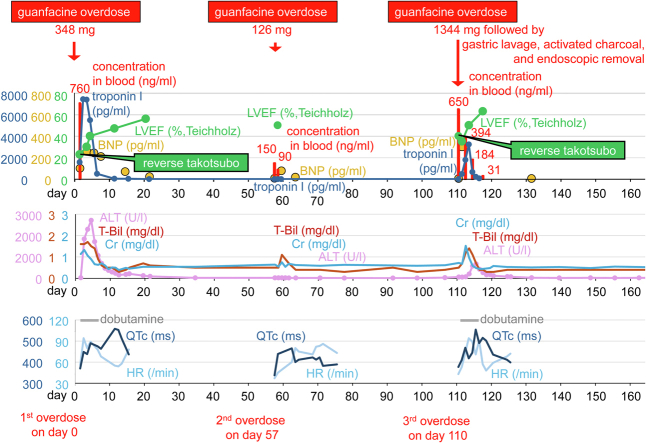

**Visual Summary:**
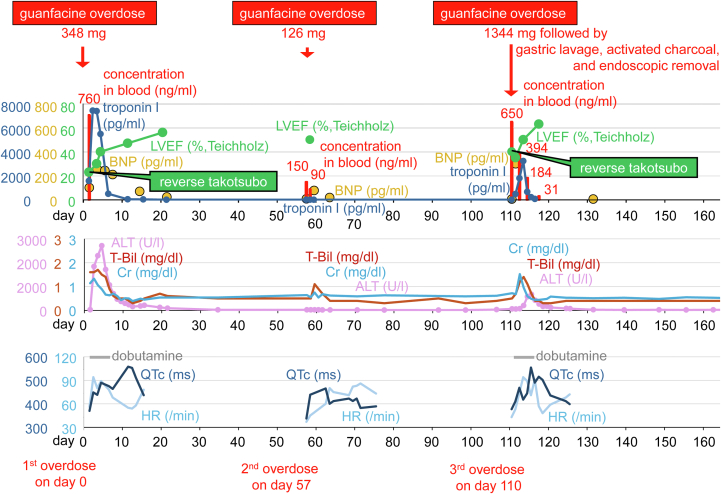
Total Clinical Course Through the 3 Overdoses ALT = alanine aminotransferase; BNP = brain natriuretic peptide; Cr = creatinine; HR = heart rate; LVEF = left ventricular ejection fraction; T-Bil = total bilirubin.

## History of Presentation

A 15-year-old girl with attention-deficit hyperactivity disorder (ADHD) and autism spectrum disorder presented to our clinic after taking 348 mg of guanfacine (the maximum prescription dose was 6 mg/d) the day before (day 0) with complaints of drowsiness. Her blood pressure was 138/96 mm Hg, pulse rate was 52 beats/min, and respiratory rate was 27 breaths/min. Her peripheral oxygen saturation (SpO_2_) was 88% on room air, and chest auscultation revealed wheezing without limb edema; therefore, she was transferred to the emergency department.

**Take-Home Messages**

• An overdose of guanfacine, a medication used for treating ADHD, can result in reverse takotsubo cardiomyopathy in a blood concentration-dependent manner.
• The cardiopulmonary time course of an extended-release guanfacine overdose spans approximately 2 weeks, with early critical respiratory failure, reverse takotsubo cardiomyopathy, and late QT prolongation.

**Figure 1:**
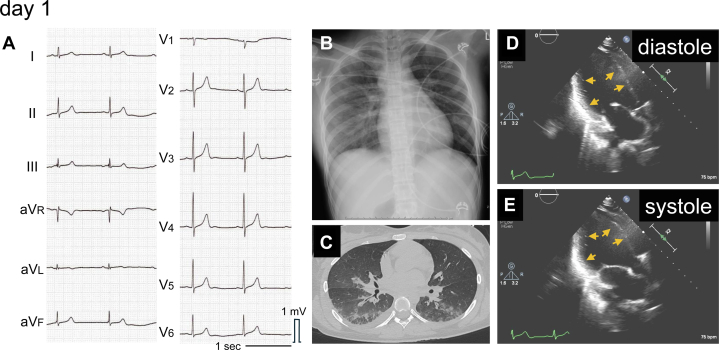
Chest Radiography, Computed Tomography, and Echocardiogram Performed on Day 1 (A) Electrocardiography showed no ST-T abnormalities. (B) Chest radiography revealed pulmonary congestion. (C) Computed tomography (CT) showed pulmonary edema and consolidation. (D and E) Echocardiography revealed left basal akinesis and preserved apical wall motion of the left ventricle. Yellow arrows indicate the endomyocardial surface.

Electrocardiography showed 56 beats/min sinus rhythm with no ST-T abnormalities (Figure 1A). Chest radiography revealed pulmonary congestion (Figure 1B). She became drowsier and more hypoxic (SpO_2_ 83% while receiving oxygen at 6 L/min) and was intubated and introduced to mechanical ventilation. Computed tomography (CT) showed pulmonary edema and consolidation (Figure 1C). Blood tests revealed elevated troponin I (1,572 pg/mL), brain natriuretic peptide (101.9 pg/mL), creatinine (1.14 mg/dL), and bilirubin (1.6 mg/dL). Echocardiography revealed left ventricular dysfunction characterized by basal akinesis and preserved apical wall motion, consistent with the morphologic characteristics of reverse takotsubo cardiomyopathy (Figures 1D and 1E, Video 1).

## Past Medical History

She had been diagnosed with ADHD and autism spectrum disorder 5 years prior and had been prescribed multiple antipsychotics, including extended-release guanfacine. She did not consume alcohol, smoke, or have a family history of heart disease or allergies. Although the patient had previously overdosed with other medications, no cardiopulmonary symptoms were observed.

## Differential Diagnosis

We diagnosed reverse takotsubo cardiomyopathy based on left ventricular wall motion abnormalities. Potential causes of severe impairment of basal left ventricular wall motion included: 1) direct inhibition of cardiomyocytes by circulating guanfacine; 2) guanfacine-dependent autonomic nerve-mediated inhibition; and 3) an emotional stress-mediated mechanism, as reported in various cases of reverse takotsubo cardiomyopathy.^1^ However, a definitive conclusion could not be reached.

## Investigations

**Figure 2:**
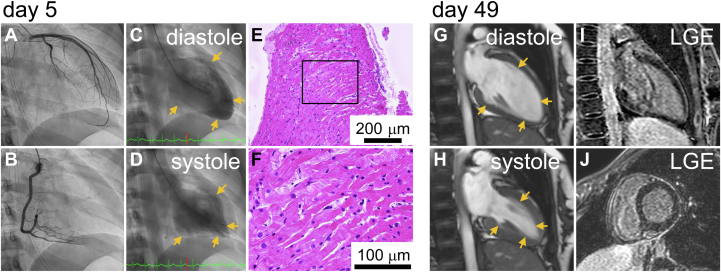
Cardiac Catheterization Performed on Day 5, and Cardiac Magnetic Resonance on Day 49 (A and B) Coronary angiography reveals no abnormalities. (C and D) Left ventriculography clearly shows wall motion typical of reverse takotsubo cardiomyopathy. (E and F) An endomyocardial biopsy shows no edema, inflammation, or fibrosis. Magnetic resonance imaging shows normal left ventricular wall motion (G and H) and no LGE (I and J). Yellow arrows indicate the endomyocardial surface. LGE = late gadolinium enhancement.

On day 2, her serum creatinine increased further (1.33 mg/dL), alanine aminotransferase levels began to rise (1,829 U/L), and her blood pressure decreased to 89/43 mm Hg. Intravenous administration of noradrenaline and dobutamine improved liver and kidney function. The brain natriuretic peptide level was 258.8 pg/mL on day 3, and it gradually decreased thereafter. On day 5, after the withdrawal of noradrenaline, cardiac catheterization was performed under intravenous dobutamine (1.3 μg/kg/min). The mean pulmonary artery wedge pressure was 17 mm Hg, and the cardiac index was 2.8 L/min/m^2^. Coronary angiography revealed no stenosis (Figures 2A and 2B). Left ventriculography demonstrated reduced basal wall motion with preserved apical wall motion, a characteristic finding of reverse takotsubo cardiomyopathy (Figures 2C and 2D, Video 2). Endomyocardial biopsy of the midventricular septum of the right ventricle showed no signs of inflammation, edema, or fibrosis (Figures 2E and 2F). The blood guanfacine concentration on day 1 was 760 ng/mL.

## Management

Left ventricular wall motion gradually improved without guideline-directed medical therapy for heart failure with reduced ejection fraction. Dobutamine was discontinued on day 6, and mechanical ventilation was discontinued on day 10. QT prolongation was observed and worsened after a week but improved by day 14. The patient was transferred to the psychiatric department. Cardiac magnetic resonance performed on day 49 showed normal left ventricular wall motion with no late gadolinium enhancement (Figures 2G to 2J, Video 3).

## Outcome and Follow-Up

**Figure 3:**
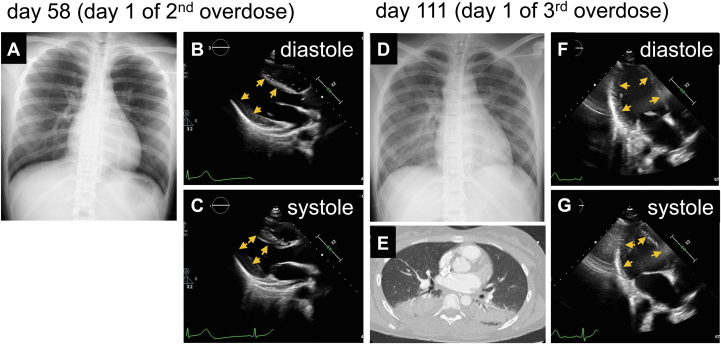
Chest Radiography, Computed Tomography, and Echocardiogram Performed on Days 58 and 111 (A) Chest radiography and (B, C) echocardiography revealed nearly normal findings. (D) Chest radiography and (E) CT revealed pulmonary congestion and dorsal consolidation. (F, G) Echocardiography demonstrated the pattern of reverse Takotsubo cardiomyopathy. Yellow arrows indicate the endomyocardial surface.

On day 57, while temporarily moving away from the hospital to undergo a school examination, she overdosed on guanfacine (126 mg), which she obtained through a social networking service, and was taken to the emergency room. She appeared slightly lethargic but did not report dyspnea. Electrocardiography, chest radiography, and echocardiography revealed nearly normal findings (Figures 3A to 3C, Video 4), except for sinus bradycardia at 37 beats/min. This second overdose did not result in heart failure symptoms or elevated troponin I levels. The blood concentration of guanfacine was lower than the first overdose (150 ng/mL on day 57).

**Figure 4:**
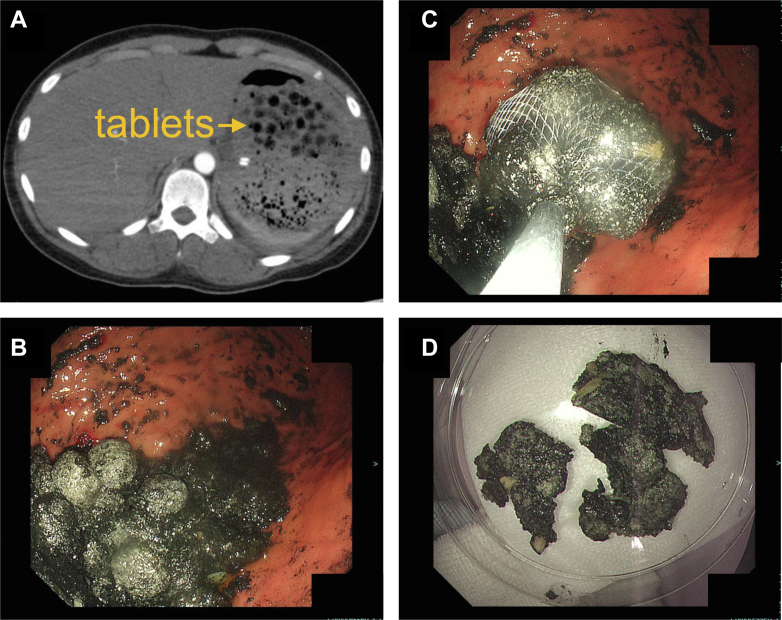
Remaining Tablets on Computed Tomography and Endoscopic Removal Performed on Day 111 (A) CT showed undissolved tablets in the stomach. (B to D) They were endoscopically removed.

On day 110, while temporarily away from the hospital, she overdosed again on guanfacine (1,344 mg) and was taken to the emergency room. Her blood pressure was 170/100 mm Hg, heart rate was 40 beats/min, and SpO_2_ was 99%. Gastric lavage and activated charcoal were administered to reduce the absorption of guanfacine. However, the following day (day 1 of the third overdose), she became drowsy and hypoxic, similar to the first overdose. The patient was intubated and placed on mechanical ventilation. Chest radiography and CT revealed pulmonary congestion and dorsal consolidation (Figures 3D and 3E). CT also showed undissolved tablets in the stomach (Figure 4A), which were endoscopically removed (Figures 4B to 4D). Echocardiography again demonstrated a characteristic pattern of reverse takotsubo cardiomyopathy (Figures 3F and 3G, Video 5), and dobutamine was initiated. The patient's condition subsequently improved, allowing for the withdrawal of dobutamine and mechanical ventilation on day 6 after the third overdose. The blood guanfacine concentration on day 110 was 650 ng/mL and decreased thereafter.

## Discussion

The lifetime prevalence of ADHD is reportedly 1% to 5%.^2^, ^3^, ^4^ Guanfacine is one of the common treatment options for ADHD. Several adverse effects associated with guanfacine overdose have been reported,^3^^,^^4^ including pulmonary edema and severe left ventricular dysfunction.^5^ However, reverse takotsubo or reverse takotsubo cardiomyopathy has not been documented in either adults or children. Although ADHD has occasionally been mentioned in reports of takotsubo cardiomyopathy, it remains unclear whether ADHD predisposes to it, or whether the association is secondary to frequent sympathomimetic use and overdose risk in this population. Drug overdose remains a significant global concern, and despite careful prescriptions by medical professionals, individuals can still obtain medications for suicide attempts through social media.^6^ Therefore, sharing information on potential adverse events and treatment consequences of overdosing on each medication is crucial for global clinical practice.

**Figure 5:**
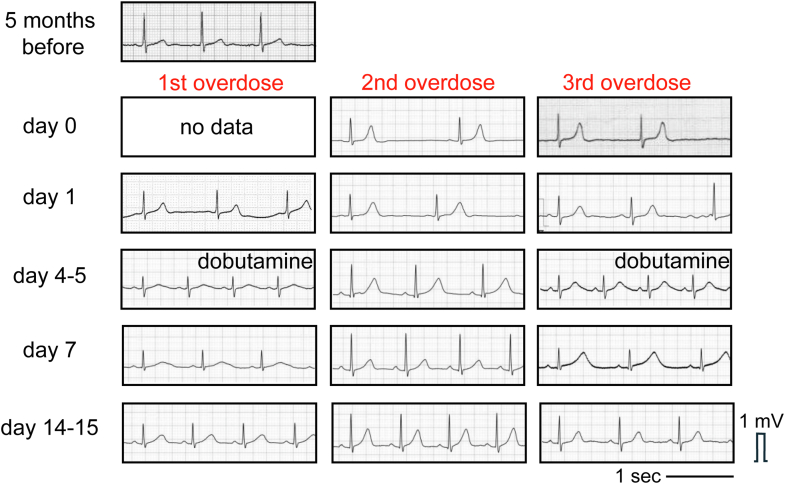
Time Courses of Electrocardiogram of the 3 Overdoses

Episodes of reverse takotsubo cardiomyopathy with troponin I elevation appear to be correlated with blood guanfacine concentrations. Initial elevations in total bilirubin and creatinine levels were observed even after the second overdose despite the absence of heart failure signs or troponin I elevation, suggesting a possible drug-induced effect. Subsequent alanine aminotransferase elevations appeared to be related to hemodynamic deterioration because they were not observed during the second overdose. QT prolongation became more pronounced as blood guanfacine concentrations declined and left ventricular dysfunction began to recover, persisting for nearly 2 weeks at most (Figure 5).

The prominent characteristics of this case were typical reverse takotsubo wall motion, reappearance, and dose dependency. Reverse takotsubo cardiomyopathy is a subtype of takotsubo cardiomyopathy.^1^^,^^7^ The mechanism behind impaired wall motion in takotsubo cardiomyopathy involves excessive adrenergic stimulation, typically induced by emotional stress, leading to an inhibitory response in cardiomyocytes via β_2_ adrenergic receptor–inhibitory Gαi protein signaling.^7^ Cardiomyocytes in the left ventricular apex have a higher density of β receptors than those in the basal left ventricle. In contrast, autonomic nerve fibers are less dense in the apex than in the base.^7^ These regional gradients could explain the varying wall motion patterns seen in takotsubo cardiomyopathy; however, the differences in the mechanisms of usual and reverse takotsubo cardiomyopathies remain largely unknown.^1^

The blood concentration–dependent time course observed in this patient suggests a role for guanfacine; however, a direct inhibitory effect on cardiomyocytes via the α2A receptor is unlikely because human cardiomyocytes are reported not to express α2A receptor messenger RNA.^8^ However, data regarding the absence of α2A receptors in cardiomyocytes are limited. Guanfacine may exert its inhibitory effects via the central and autonomic nervous systems; however, it remains unclear whether these neural mechanisms caused by guanfacine are strong enough to induce akinesis at the base of the left ventricle.

Reverse takotsubo cardiomyopathy has been reported in various stress scenarios, including overdose and suicide attempts.^1^^,^^9^ Thus, emotional stress–mediated mechanisms other than guanfacine may have contributed to this case. However, the absence of reverse takotsubo cardiomyopathy during the second overdose with a relatively low blood guanfacine concentration cannot be explained. Therefore, we were unable to distinguish between the direct and indirect effects of guanfacine and those caused by emotional stress.

When considered alongside the single prior case that quantified guanfacine concentrations at the time of acute respiratory failure, the plausible threshold for severe left ventricular dysfunction causing acute respiratory failure may lie between 150 and 300 ng/mL. This estimate likely varies with individual susceptibility and concomitant medications, and additional cases are needed. Notably, QT prolongation related to blood guanfacine concentration has also been reported in a previous case.^10^ However, in our case, QT prolongation became more prominent after the blood concentration decreased, and the maximum blood concentration (760 ng/mL) was higher than that reported in the previous case (13 ng/mL).^10^ Even if the mechanism is not directly related to guanfacine itself and is instead secondary to changes in the electromechanical characteristics of cardiomyocytes, it is vital to recognize this late critical abnormality.

## Conclusions

In a 15-year-old girl with ADHD, repeated overdose of extended-release guanfacine caused reverse takotsubo cardiomyopathy, accompanied by respiratory failure, within 2 days of intake in a blood concentration-dependent manner. This was followed by the late exacerbation of QT prolongation, lasting as long as 2 weeks.

## Funding Support and Author Disclosures

The authors have reported that they have no relationships relevant to the contents of this paper to disclose.

## Appendix

Video 1 Echocardiography on Day 1

Video 2 Left Ventriculography on Day 5

Video 3 Cine Magnetic Resonance Imaging on Day 49

Video 4 Echocardiography on Day 58 (Day 1 of Second Overdose)

Video 5 Echocardiography on Day 111 (Day 1 of Third Overdose)
